# Supplementary material for: Ultrasonic Microbubble Cavitation Deliver Gal-3 shRNA to Inhibit Myocardial Fibrosis after Myocardial Infarction
Source: Pharmaceutics. 2023 Feb 22;15(3):729. doi: 10.3390/pharmaceutics15030729 (PMC10051524; doi:10.3390/pharmaceutics15030729)
Supplement: Supplementary file 1 [file pharmaceutics-15-00729-s001.zip › pharmaceutics-2173451-supplementary.pdf]

Supplemental Information

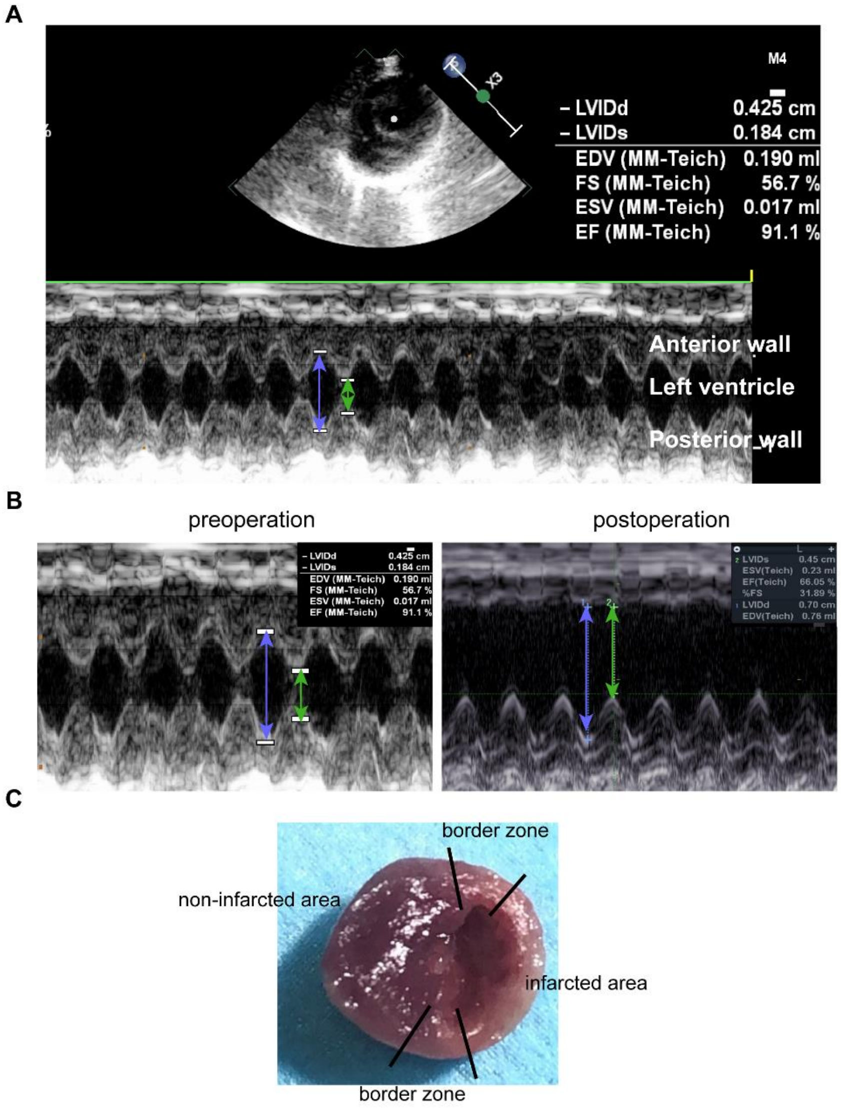

Figure S1. The measurement of cardiac function by M-mode echocardiography and schematic diagram of different regions in MI heart.

(A) A raw measurement image M-mode image in a normal rat. (B) Representative M-mode images of MI rats at preoperation and postoperation phase. The blue line showed the measurement of LVIDd, the green line showed the measurement of LVIDs. (C) Schematic diagram of different regions in MI heart. The infarcted area, border zone, and non-infarcted area are illustrated as indicated

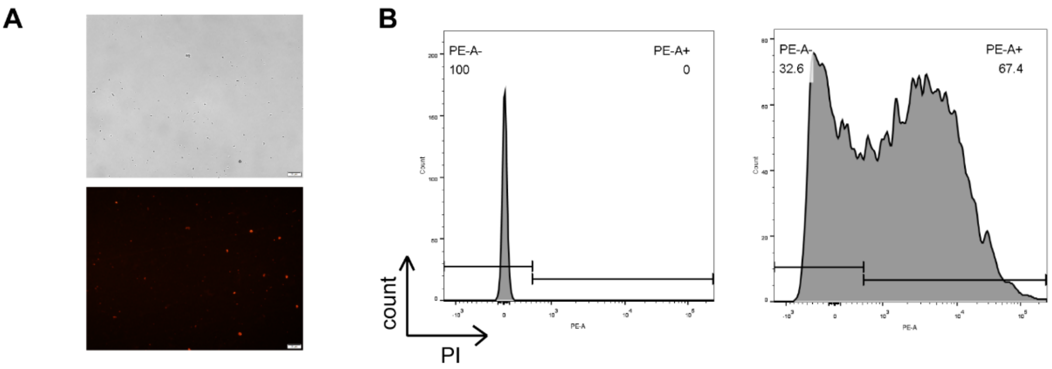

Figure S2. The binding rate between Gal-3 shRNA and CMBs

(A) PI staining of Gal-3 shRNA / CMBs (B) Flow quantitative analysis of Gal-3 shRNA / CMBs

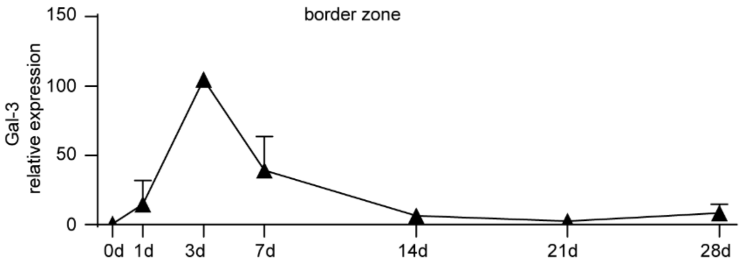

Figure S3. Gal-3 mRNA expression of border zone post MI

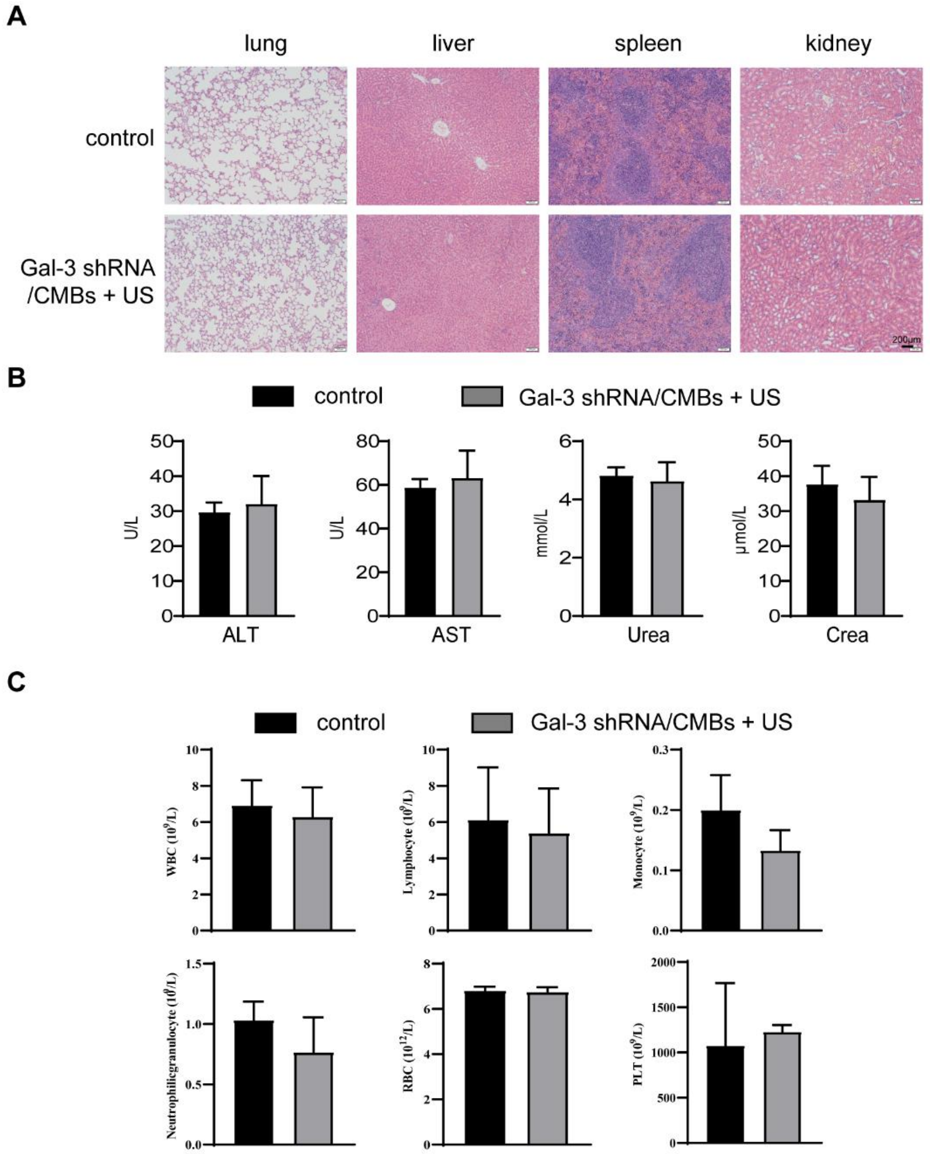

Figure S4 Toxicity evaluation in vivo.

(A) HE staining of lung, liver, spleen and kidney tissue sections. (B) serum levels of ALT, AST, Urea and Crea (C) main indexes in examination of blood routine on day 21 ($n = 3$, $p > 0.05$)
